# LightBox: A multiwell plate illumination system for photoactive molecule characterization

**DOI:** 10.1002/jbio.202000481

**Published:** 2021-02-22

**Authors:** A. D. Bounds, R. D. Bailey, C. T. Adams, D.C. Callaghan, J. M. Girkin

**Affiliations:** ^1^ Centre for Advanced Instrumentation, Department of Physics Durham University Durham DH1 3LE UK; ^2^ Department of Biosciences Durham University Durham DH1 3LE UK

**Keywords:** bio‐instrumentation, bio‐photonics, photoactive molecules, photodynamic therapy

## Abstract

Multiwell plates (MWPs) are the workhorses of the life sciences. However, biophotonics research with MWPs is limited, in part due to the lack of equipment suitable for photo‐irradiation of photoactive molecules in a MWP‐suitable, high‐throughput manner, either commercially or through open‐source MWP systems. Here we present “LightBox”, a calibrated controllable MWP illumination system with broad applications including screening of photoactive molecules and characterization of photocatalytic chemicals. LightBox is a high intensity, accurately controllable, uniform illumination system designed for MWPs with electronics and a control unit that provides a simple and intuitive interface. LightBox can reach intensities of 0.23 mW/mm^2^ at wavelengths of 405 nm with variance between well sites of <5%. The usefulness of LightBox is demonstrated by assessing the IC50 of a photosensitizing compound using a live/dead assay following simultaneous irradiation of the sample at a range of concentrations, eliminating uncontrolled variables between concentrations and drastically increasing assessment speed.
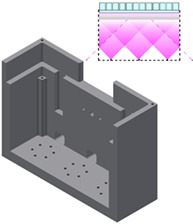

## Graphical abstract

Multiwell plates (MWPs) are ubiquitous in life sciences, but are underutilized in biophotonics due to limited suitable MWP instrumentation. Here we present LightBox, a calibrated, controllable highly uniform and high power MWP illumination system suitable for screening drugs for photosensitivity, assessing photocatalysis effects and characterizing effectiveness of photodynamic therapeutics in a reliable and high‐throughput manner. LightBox reaches intensities of 0.23 mW/cm^2^ at 405 nm, with variance of less than 5%.

**Abbreviations**
PDT: photodynamic therapy
PWM: pulse width modulation.
RPi: Raspberry Pi

## 1 INTRODUCTION

Multiwell plates (MWPs, also known as microplates) are ubiquitous across biology, chemistry, pharmaceutical research and more. These are flat plates with multiple “wells” that are used as small test tubes for analytical research and clinical diagnostic testing. They are used for a very wide range of applications including compound preparation,^1^ high‐throughput screening,^2^ sample collection, cell culture growth^3^ and combinatorial chemistry.^4^ Common usages include enzyme‐linked immunosorbent assays (ELISA)^5^ and polymerase chain reaction (PCR) assays^6^ such as those used to detect the presence of the SARS‐CoV‐2 virus. The global MWP instrumentation and supplies market exceeded $4.5Bn in 2017.^7^

From their first use in the 1950s^8^ MWPs have been critical in the expansion of the pharmaceutical sector, allowing high‐throughput screening of drugs and chemical compounds and high‐throughput assays. MWPs are standardized through ANSI/SLAS standards, which ensures uniformity in dimensions and well positions.^9^ Typical well layouts are in a 2:3 or 3:4 rectangular matrix with 6, 12, 24, 48, 96, 384 or 1536 wells and varying volume capacities per well. Different well bottom shapes and depths are available, and coatings can optimize adhesion. The uniformity of MWPs has enabled a massive market for MWP products such as plate readers, robotic plate movers and stackers, liquid handling robots, plate washers, thermal sealers and de‐sealers, and incubators. Biophotonics plays a key role in many of these products (e.g. fluorescence readers, spectrophotometric readers). A range of MWP colors are available, including MWPs transparent at a range of different wavelengths and MWPs with transparent bottoms and opaque well sides to best facilitate these biophotonic applications. The diverse range of products is key to high throughput testing and screening to meet the wide variety of applications that MWPs offer.

This diversity of MWP products is reflected in the emergence of open‐source MWP systems. Open‐source platforms already enable fluorescence imaging,^10^, ^11^ spectrophotometry,^12^ robotic liquid handling^13^, ^14^ and turbidostats^15^ and there are a growing number of projects applying these platforms to MWPs. Open‐source plate readers offer a 10‐fold reduction in price compared to commercial systems,^16^ colorimetric readers have been successfully benchmarked against FDA‐approved ELISA tests^17^ and low‐power, well‐specific MWP illumination systems are available for optogenetic stimulation.^18^, ^19^ Hybrid open‐source approaches also exist, for example, modifications to commercial plate readers that enable controlled well illumination for photobiology experiments.^20^ Much of this emergent field of open‐source technology is thus orientated around biophotonics MWP systems, reflecting the desire for high‐throughput scalable approaches to biophotonics research. Light is used as the trigger for photodynamic therapy (PDT), with the level of reactive oxygen species (ROS), and thus phototoxicity, a complex function of photosensitizer concentration, illumination properties (wavelength, intensity, exposure time etc.) and cell conditions.^21^, ^22^ Light can also be used to control reaction rates in the presence of photocatalysts, again with a complicated relationship between illumination parameters and experimental parameters.^23^ New drugs must undergo screening for photodegradation and to assess potential photosensitivity that would make them ill‐suited to use.^24^ All of these light‐activated solutions and hazards require extensive screening and testing of the effects that new molecules and drugs have on cell cultures whilst under illumination by highly uniform and controllable illumination in controlled environments.

Whilst the commercial sector provides highly engineered and optimized MWP systems and devices, they are poorly suited to high‐throughput, high‐power, highly uniform MWP illumination without significant modification. Some open‐source systems enable controlled low‐power illumination of specific wells within MWPs for optogenetics,^18^, ^19^, ^20^ but there are few high‐throughput highly uniform MWP illumination systems either commercially or through open‐source platforms. Here we present a multiwell plate photo‐irradiation system, “LightBox”, which is compatible with MWP dimensions. LightBox offers calibrated highly uniform illumination of MWPs with controllable, repeatable and programmable illumination times and intensities and is suitable for a very broad range of applications including characterization of photodamage, effectiveness of photosensitizers and photodynamic compounds, and assessment of photocatalysis. The system presented here uses 405 nm light with intensities of up to 0.23 mW/mm^2^ to match transition wavelengths in target PDT compounds, but the system is suitable for a wide range of wavelengths. The system interface utilizes a Raspberry Pi (RPi) configured as a WiFi router with a browser‐based interface, allowing remote control of the system using commonly available WiFi‐enabled electronics such as smart‐phones or tablets. All of the computer software and hardware drawings are available on the GitHub repository for the device.^25^

To verify the efficacy of the system, LightBox has been used to characterize the effectiveness of a photosensitive compound, demonstrating that LightBox can be used to rapidly assess the relationship between cell viability, cell death, dark toxicity and compound concentration. LightBox thus has a broad range of applications including screening drugs for photosensitivity, assessing photocatalysis effects and characterizing effectiveness of photodynamic therapeutics, all in a reliable and high‐throughput manner.

## 2 METHOD

**FIGURE 1:**
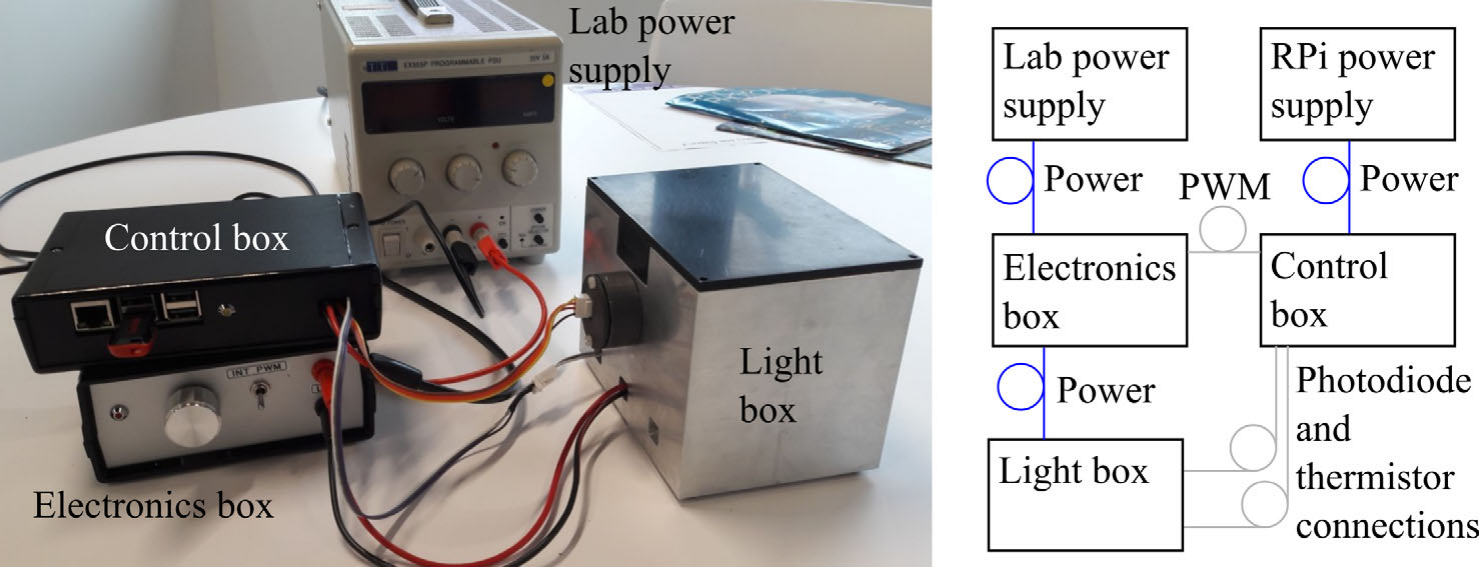
The light delivery box and control elements (left). Right, the connections between the different elements of the system are illustrated

The complete LightBox system consists of a light delivery box, an electronics unit housing power electronics, a control unit containing the interface electronics and LED and interface power supplies, shown in Figure 1. The original system was designed to operate around 405 nm at the peak activation wavelength of the compounds originally under investigation. The details described below are, however, applicable for any wavelength of high power LED from the deeper UV through to the near infrared providing that the LEDs have appropriate spatial emission profiles.

### 2.1 Light delivery box

The light delivery box consists of an aluminum housing designed to house six high power LEDs with ridges to allow the mounting of a standard MWP above the LEDs. It is preferable to illuminate the MWP from underneath to prevent significant reflections from within the MWP wells. In earlier iterations such reflections were found to be a cause of local intensity “hot spots” within the wells.

**FIGURE 2:**
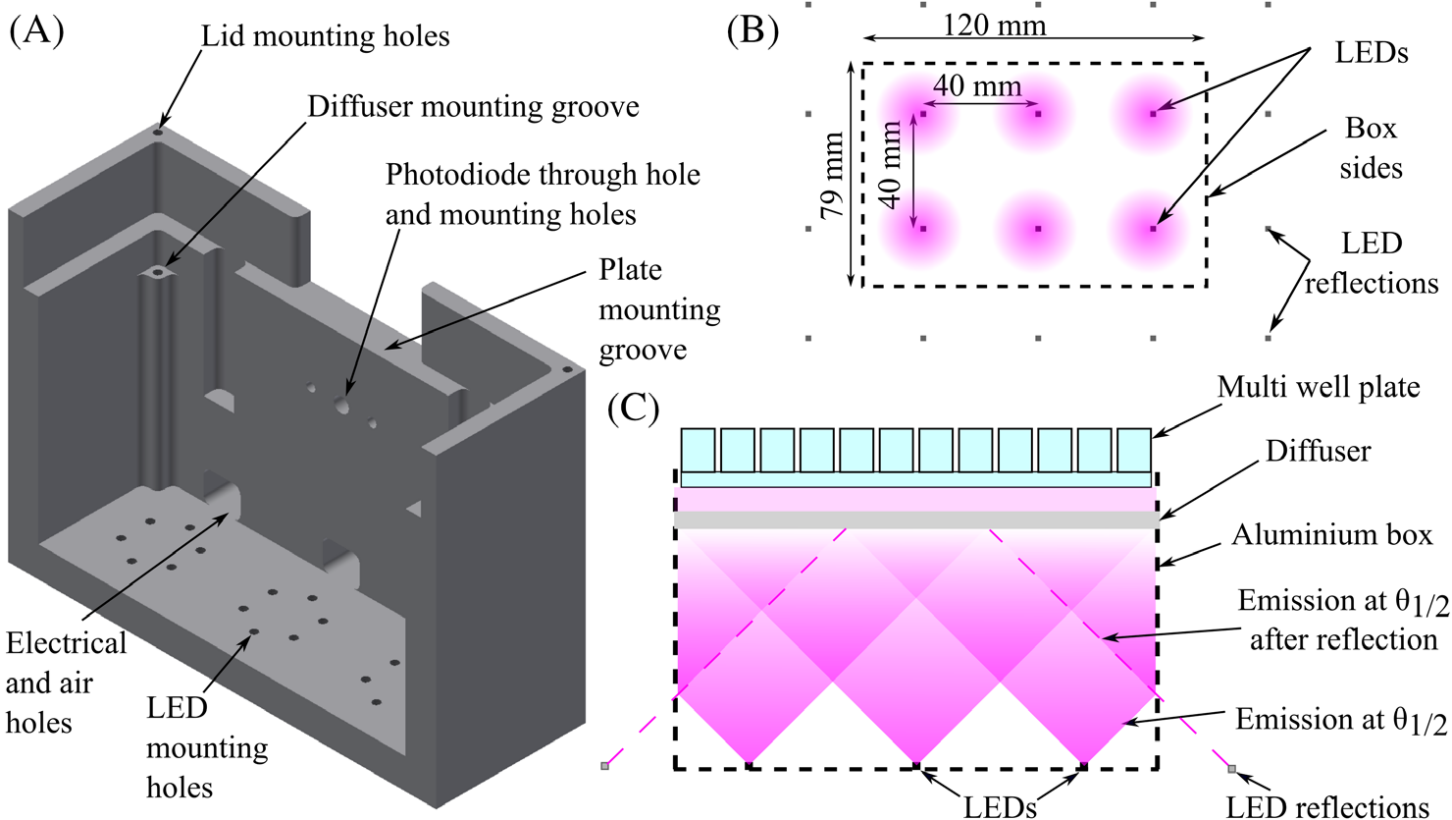
A, Half‐cut through the light delivery box, illustrating the key components of the light delivery box. B, LED layout. Uniform positioning of LEDs and reflections from the light delivery box walls ensure the LED positioning matches the light delivery box layout. C, The emission cone from the LEDs, the reflections from the box sides, and the height of the diffuser from the LEDs ensures diffuser is uniformly illuminated. It should be noted that whilst this figure illustrates the *θ*_1/2_ emission profile of the LEDs, the actual emission profile smoothly falls with angle

A high priority is to ensure uniform illumination, with a target intensity variation of 5% of output power across the entire MWP. To this effect, LEDs with wide angles of emission are used (Opulent Americas 416‐LST101G01UV04, emission output 930 mW, output angle *θ*_1/2_ = 130°). The LEDs are screwed directly onto the aluminium housing with a layer of thermal paste to ensure good conductivity from the LEDs to the light delivery box. This prevents the LEDs overheating and ensures that the heat generated inside the enclosure is conducted to the external face to minimize the risk of sample heating. The light delivery box was machined from a solid block of aluminum to maximize the thermal capacity of the system and minimize system heating, although to reduce machining time it is also possible to machine the box sides and base separately. Aluminum is chosen as it has high reflectivity across a broad spectral range. The LEDs are evenly spaced in two rows of three LEDs spaced 40 mm apart and 19–20 mm inset from the edges of the light delivery box. When considering reflections from the sides of the housing and the emission properties of the LEDs, this creates an array with uniform spacing that closely matches the internal dimensions of the light delivery box, which itself closely matches the dimensions of the standard MWP as shown in Figure 2.

A plastic diffuser (Engineering and Design Plastics Ltd, 3 mm clear acrylic frost sheet) is positioned 60 mm above the LEDs to improve uniformity, remove any residual pattern in the light and randomize photon direction close to the MWP. The separation of the diffuser from the LEDs is chosen such that the LED light cone at the diffuser has a diameter (128 mm) roughly equal to the size of the MWP well region (121 mm). This ensures that all wells are illuminated by multiple LEDs, reducing sensitivity to any power or wavelength variation between LEDs.

The plastic diffuser can be screwed into the light delivery box to prevent movement as small supporting posts sit in the light delivery box. The MWP is positioned 10 mm above the diffuser and sits on a second ledge. Grooves either side of the MWP allow easy access to lift and place the MWP in and out of the light delivery box without tipping or disturbing the sample.

Access holes at the bottom of the light delivery box allow electrical connections to be made to the LEDs and allow ventilation. These access holes also allow a connection to be made with a temperature sensor (Analog Devices TMP37FT9Z) mounted inside the light delivery box that enables continuous temperature tracking of the light delivery box. A second monitoring system for the light delivery box is a photodiode (Osram BPX65) inserted into the light delivery box to monitor output power; two tapped holes either side of the photodiode hole allow a photodiode mounted on a simple low‐pass zero‐bias PCB to be screwed to the light delivery box level with the light delivery box internal walls.

An opaque lid 3D printed in black PLA protects the user from exposure to light and prevents reflections from the lid back to the sample. If required, an interlock switch could be added to ensure that illumination is disabled whenever there is access to the light delivery box.

### 2.2 Electronics

The LEDs are powered by an electronics box connected to the light delivery box. Housing the electronics separately from the LEDs and wet sample prevents fluid ingression onto the electronics and minimizes sample perturbation by heat sources such as the drive electronics.

The LEDs are powered in series, as this constant current configuration results in less variation in output power between different LEDs compared to a constant voltage, parallel connection configuration. A consequence of this is that a single LED failing is more obvious as it will prevent emission, which is preferable as it reduces the likelihood of device misuse.

Accurate control of the illumination intensity is required for quantitative studies. Simple changes to the current to control output power are not an appropriate method to vary illumination power as LED emission spectra can vary with current and the relationship between LED current and LED output power is not necessarily linear. Instead pulse‐width modulation (PWM) is used to modulate the light level delivered to the sample using a time‐proportioning control technique. The LED drive current is modulated at a frequency of 1 kHz, which is much slower than typical chemical processes but much faster than the response of the human eye and typical exposure times. PWM details are found in Supporting Information 1. Maximum output from the electronics box to the LEDs is 25 V at 1 A, thus allowing up to 4.2 V across each LED, well above the 3.4 V required by each LED operating at 405 nm. The drive voltage can be varied to suit the activation voltages of different LEDs and different wavelengths. The electronics box is powered by a laboratory power supply (Aim TTi, EL303R) with a supply of up to 30 V at 1 A.

### 2.3 Control

**FIGURE 3:**
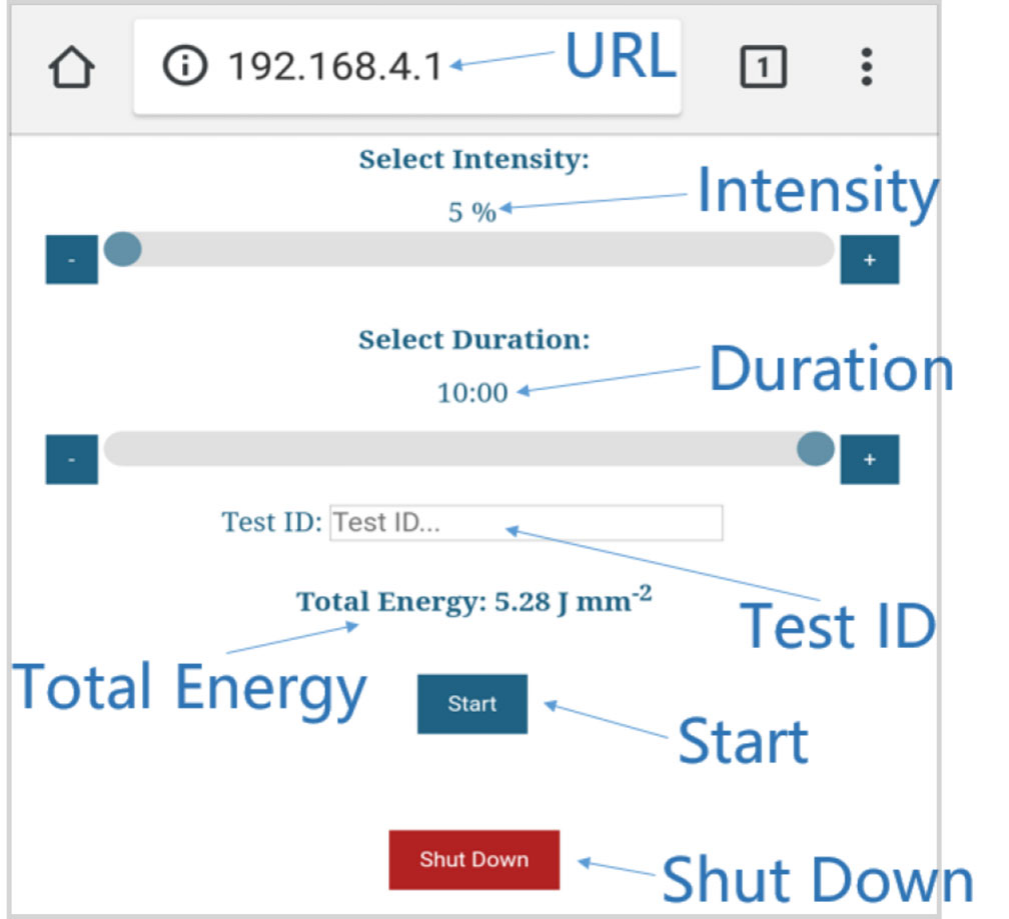
Control interface. The user navigates to the IP address in a browser and sets the exposure intensity and duration using a slider interface. The Test ID is required and enables the user to distinguish between experimental datasets in saved files

The system is controlled and monitored using a Raspberry Pi (Raspberry Pi 3 Model B+) connected to the electronics box and the light delivery box. The RPi is configured to act as a WiFi router. When the system starts, the control program is started, which acts as a local web host. This allows any WiFi‐enabled device with a web browser to navigate to a browser‐based control interface when it is connected to the RPi's WiFi network. Flashing LEDs indicate when the RPi is ready to control the system. Through this interface, shown in Figure 3, the LightBox intensity and exposure time can be controlled in intervals of 5% of peak intensity and 10 second steps up to 10 minutes using a slider interface. These values are the smallest increments required (though could be altered by simple changes to the program if required) and result in increments large enough to make the slider interface easy to use. The intensity is controlled using PWM of a digital output from the RPi to the electronics box via a BNC cable. The interface also requires the sample name to be recorded with the data and start time.

The control system also monitors the temperature sensor and photodiode mounted in the light delivery box. Box temperature and illumination intensity are recorded to a comma‐separated variable (CVS) file on a USB stick inserted into RPi within the control box. Readings are taken every 5 seconds and saved with the sample name and the date and time that the sequence begun. This allows the conditions that a sequence was run in to be checked and logged into any other data and sample processing software used in the experiment. The control box monitoring system is described in Supporting Information 2.

## 3 RESULTS

**FIGURE 4:**
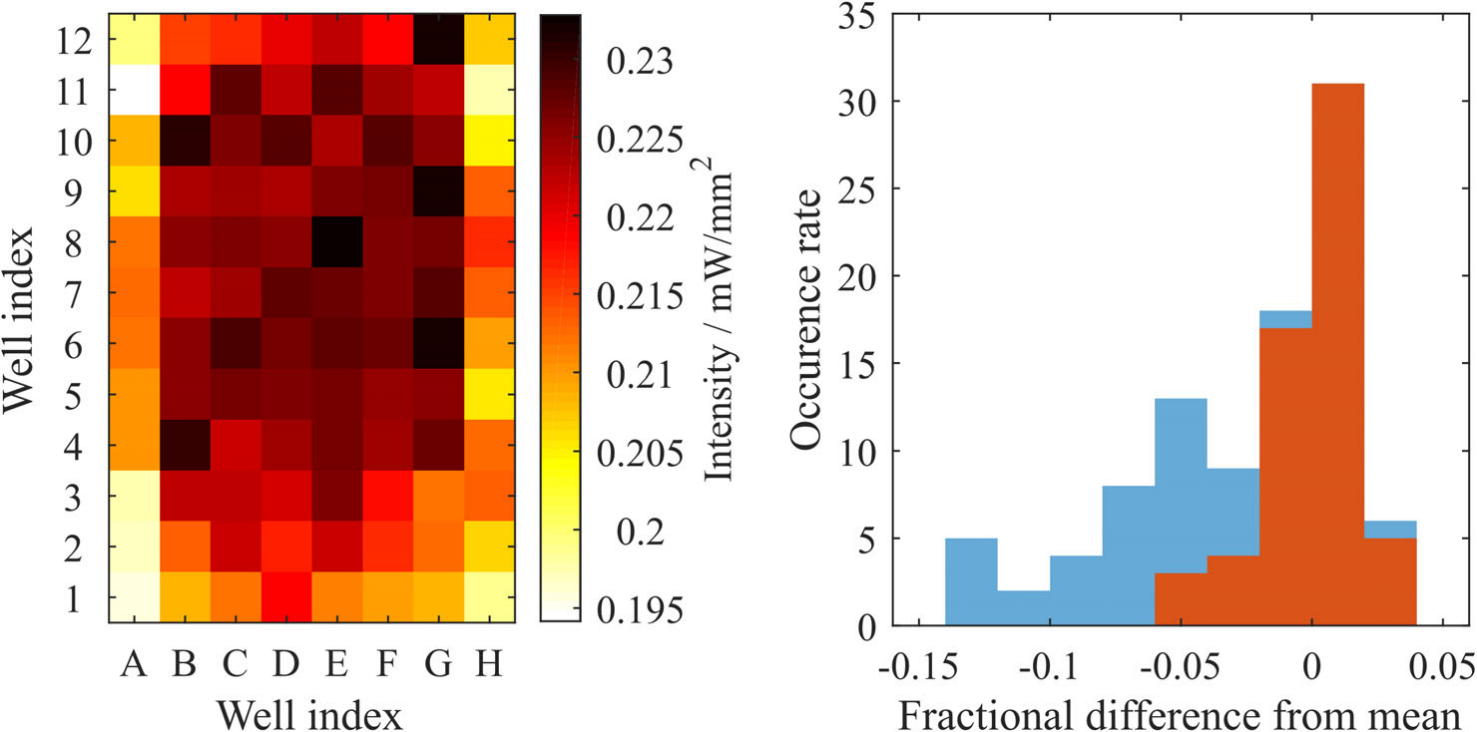
A, Intensity uniformity colormap. Clearly the edge wells are weaker than the average, resulting in significant non‐uniformity. Histogramming the intensity B, we see that by excluding edge wells (blue) from all other wells (orange) the variation in intensity can be reduced to less than 5% of the mean intensity. Outer wells have been excluded from the mean intensity

The key requirements of this system are high illumination intensity, high illumination uniformity across a MWP, controllable exposure and intensity, and ease of control. Illumination intensity at the well plate was measured and mapped with a photodiode (Osram BPX65) mounted on a simple low‐pass zero‐bias PCB circuit. Each well of a 96 well plate was measured three times by inserting the photodiode into each well. The mean illumination intensity at full illumination power was measured as 0.23 mW/mm^2^, sufficient to generate irradiance of 0.14 J/mm^2^ in 10 minutes. Intensity variation between wells is shown in Figure 4.

Clearly, outer wells display reduced illumination intensities. However, this variation is structured and is not as severe a problem as random intensity variation between wells. Excluding the outer wells, we observe high levels of uniformity, with all inner wells operating with illumination intensities within 5% of the mean as shown in Figure 4(b).

Variation of illumination intensity as a function of set intensity was measured by observing the output power with a zero‐bias low‐pass photodiode in 25 wells as a function of set intensity. Fitting a linear curve to the intensity as a function of set intensity, a reduced chi‐squared of *χ* ^2^ = 1.06 was measured, confirming the reliable control of intensity exposure.

One concern is thermal effects caused by power dissipation from the LEDs. Running the box at the maximum intensity and exposure time (10 minutes), a box temperature change of less than 2°C was observed (measured with both the inbuilt thermistor and a separate thermocouple). This is within acceptable limits, particularly given that typical run times are less than 10 minutes.

Finally, the LED emission spectrum was measured with a spectrometer (Ocean Optics Flame, calibrated using a mercury emission line [546.1 nm] and a 632.8 nm Helium Neon laser). The peak emission wavelength is 405.2 ± 0.1 nm with a spectral width (FWHM) of 15.5 nm. All LEDs are within 1 nm of this peak wavelength and no dependence on PWM duty cycle was observed.

## 4 DISCUSSION

A key requirement of this system is the uniformity of the illumination light. A maximum variation of 5% is well within acceptable limits and is below the 20% variation in intensity that is acceptable within clinical interventions. This variation of 5% is obtained by excluding the outer wells in a 96 well plate. Outer wells of multi‐well plates often exhibit slightly different behavior to inner wells as they have higher environmental sensitivity, it is therefore commonplace to exclude outer wells as we do here.

Typically, variability in MWP assessments is characterized by the coefficient of variation (CV), with inter‐assay CV of less than 15% and intra‐assay CV of less than 10% deemed generally acceptable. The coefficient of variance accounts for multiple sources of variance. A maximum well‐to‐well variance of 5% is therefore well within acceptable limits. Care must be taken with direct comparisons between this system and the coefficient of variance obtained with other systems involving illumination as existing plate readers and illumination methods typically illuminate each well individually in sequence, rather than simultaneous illumination of the entire sample. However, we do note that a maximum variance of 5% across a 96 well plate (excluding outer wells) is comparable to measurements from the open‐source plate reader,^16^ which has a CV of 3.1% and a maximum variance of 8.6% with a 96 well plate.

Simultaneously illuminating the sample has two notable advantages. Firstly, it eliminates a potential variable that exists with sequential illumination for processes with high sensitivity to when the sample is illuminated. Secondly, and more importantly, it ensures that all of the sample in the well experiences similar illumination. Typically, in plate readers, a collimated beam illuminates the center of the well, resulting in a varying illumination profile across the sample. This collimated beam is kept small to avoid illumination of neighboring wells (cross‐talk) that could interfere with measurements. However, when the illumination light may permanently modify the sample (as is the case when assessing photodynamic therapy compounds, photosensitization, photocatalysis, optogenetics etc.), this can result in undesirable variation across a single well. Here, we avoid this issue by ensuring uniform illumination across the well.

Whilst a detailed investigation into the source of the reduced intensity experienced by outer wells has not been conducted, we note that reflection from the sides of the box is used to simulate the effect of a larger array of LEDs. We also note that reflectivity typically falls as incidence angle rises. Reflections at glancing angles to the box that will preferentially illuminate outer wells will therefore be more suppressed by reduced reflectivity, potentially resulting in this reduced intensity experienced by the outer wells. The effect will be significantly less pronounced in well plates containing fewer wells where their inherently larger size will reduce the local variation in intensity.

The achievable intensities of 0.23 mW/mm^2^ are well below those used in medical interventions (which may reach 10 mW/mm^2^ when focused onto a small area with short pulses,^26^ but are well‐suited to characterization of samples in MWPs, where there is little absorptive material between the light source and the photosensitizer and where uniform illumination across a wide area is required. These intensities also exceed those achievable with comparable open‐source systems: Gerhardt et al^18^ achieve intensities on the order of 0.005 mW/mm^2^ whilst illuminating all wells of a 24 well plate simultaneously (with illumination that can be controlled for each well), whilst Richter et al^20^ achieve intensities of up to 0.1 mW/mm^2^ illuminating each well sequentially. These systems are designed for different illumination profiles (exposure time, wavelength) within the same plate and may experience non‐uniform illumination across each well as described above, rather than the high‐throughput highly uniform, high power system presented here.

**FIGURE 5:**
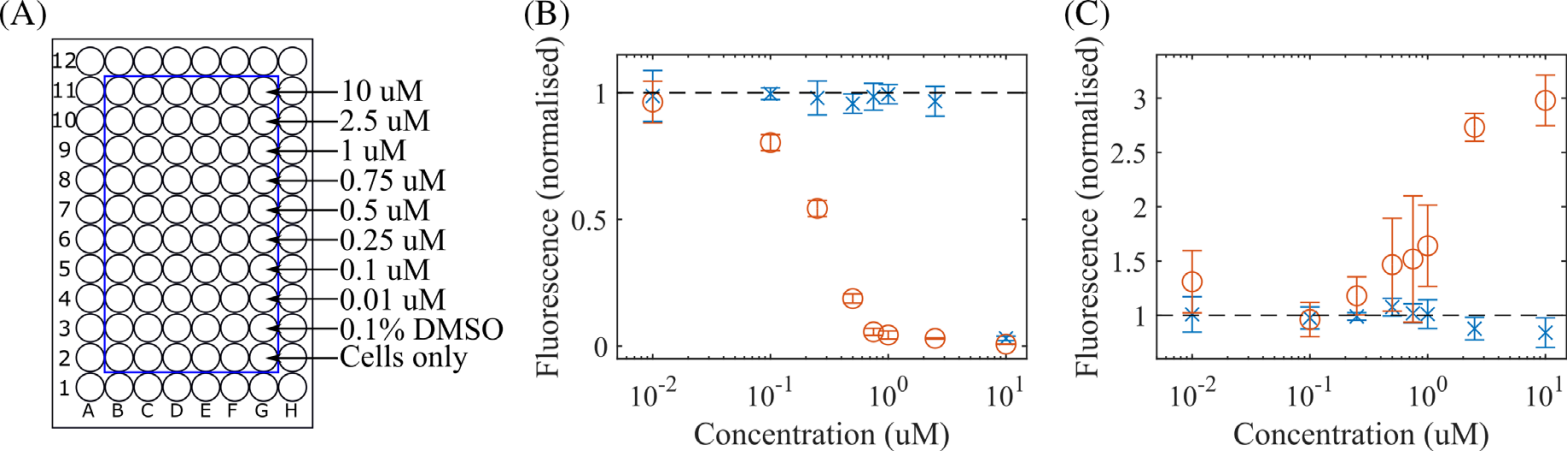
Assessment of photosensitizing compound effectiveness. A, Sample set‐up. Outer wells are not used, the 10 inner rows each contain different compound concentrations and controls, with 6 repeats of each, allowing simultaneous illumination of multiple samples to avoid uncontrolled variables between different compound concentrations. B, Fluorescence from FDA stained HACAT cells. Orange rings represent illuminated samples; blue crosses represent non‐illuminated samples. At compound concentrations of 0.1 μM and higher, FDA fluorescence is reduced from irradiated samples, indicating a reduction of hydrolysis and cell viability that is not observed in non‐illuminated samples, other than dark toxicity observed at the highest compound concentration. C, Fluorescence from PI stained HACAT cells. At compound concentrations of 0.5 μM and higher, PI fluorescence begins to rise, indicating the presence of DNA outside of cell membranes. Black dashes indicate fluorescence levels in the absence of the photosensitizing compound

The utility of the system is demonstrated in Figure 5, which shows an assessment of the effectiveness of a photosensitizing compound developed by LightOx Ltd. This uses a PI/FDA assay, a cell viability assay where Propidium Iodide (PI) assesses cell death and Fluorescein diacetate (FDA) assesses viable cells. PI fluorescence is wavelength‐shifted and enhanced by a factor of 20–30 when binding to DNA, this increase in fluorescence is used to detect cell death. FDA is a non‐fluorescent molecule, which is hydrolyzed to fluorescent fluorescein in live cells, this fluorescence thus indicates living cells as only living cells hydrolyze FDA.

A cell culture was prepared combining HACAT cells with the LightOx compound in varying concentrations from 0.01 μM to 10 μM with controls of cells only and cells in a 0.1% dimethyl sulfoxide (DMSO) solution. Cell culture and fluorescence assessment details are found in Supporting Information 3. The cell culture and photosensitizing compound were added to the inner wells of a 96‐well MWP. The 60 inner wells comprise 10 rows of 6 wells, with each row used for a different concentration of photosensitizer compound with six replicates of each (illustrated in Figure 5A). This system thus allows 60 wells to be simultaneously illuminated with high, uniform intensities in identical conditions to precisely characterize photosensitizer effects. The cells were illuminated using the LightBox for 300 seconds at 100% intensity (0.23 mW/mm^2^) prior to washing and staining with FDA or PI, and were then inspected using a fluorescence plate reader. The same process was repeated with a control that was not illuminated by the LightBox.

Results are shown in Figure 5B,C. The FDA clearly shows a decay in fluorescence in the irradiated samples that does not occur in the control (non‐irradiated) samples, starting at concentrations of 0.1 μM and rising until fluorescence is almost completely suppressed at concentrations of 1 μM. This indicates a reduction in cell viability that occurs with photosensitizer illumination. At the highest concentration of photosensitizer (10 μM) dark toxicity is also observed in the sample that was not illuminated.

Cell death is verified with the PI stains, which show an increase in fluorescence from photosensitizer concentrations of 0.5 μM and greater when the sample is illuminated with the LightBox. This fluorescence increase indicates an increase in cell membrane disorder, indicating cell death.

The compound concentration threshold at which FDA fluorescence falls is different to the threshold at which PI fluorescence increases, as FDA indicates cell viability as indicated by hydrolysis, whilst PI indicates cell death as indicated by DNA outside of cell membranes. This also results in dark toxicity not being observed with PI.

From the reduction in cell viability and increase in cell death identified by FDA and PI respectively, the effectiveness of the photosensitive compound can be assessed, demonstrating the thresholds at which cell viability is impacted and cell death occurs, as well as allowing the identification of when dark toxicity occurs.

This thus demonstrates the utility of the LightBox, allowing the simultaneous illumination of 60 wells of a 96‐well plate under identical conditions with a controllable uniform light source. The ability to illuminate so many samples simultaneously under identical conditions allows multiple concentrations to be assessed simultaneously, with repeats, eliminating a wide range of variables that could occur if each sample needed independent illumination and reducing experiment time.

Alternate applications include assessment of degradation of fluorescence quantum yield with illumination and study into the effects of exposure time and intensity on effectiveness of PDT. An alternate version of the LightBox has been developed using 365 nm LEDs with similar emission profiles and achieving similar uniformity of illumination.

## 5 CONCLUSION

We have presented LightBox, a multiwell plate illumination system for characterizing photoactive molecules in a reliable high‐throughput manner. The system has a broad range of applications including screening drugs and molecules for photosensitivity, characterization of photocatalytic chemicals and assessment of photodynamic therapeutic compounds. LightBox illuminates a standard‐dimension multiwell plate with controllable exposure times up to 10 minutes and intensities up to 0.23 mW/mm^2^, with automatic intensity and temperature monitoring and remote control. LightBox uses 405 nm light, but the system is designed to take any LEDs with wide emission angles. The utility of the LightBox is shown with the assessment of the effectiveness of a photosensitizer, where the LightBox allows the characterization of the photosensitizer concentration at which cell viability is impacted and cell death occurs. This characterization was performed with all repeats and photosensitizer concentrations simultaneously illuminated, minimizing uncontrolled variables between concentrations and experimental repeats to improve data quality and reducing experimental run‐time.

## CONFLICTS OF INTEREST

C. T. Adams and D.C. Callaghan are employees of LightOx Ltd, which is commercializing a second‐generation version of the LightBox system. In addition, C. T. Adams is an inventor on a patent application regarding the second‐generation system. A. D. Bounds, R. D. Bailey and J. M. Girkin declare no conflicts of interest.

## Supporting information


**Appendix S1**: Supporting Information

## Data Availability

In addition to the GitHub repository, the data presented in this paper are available online at 10.15128/r2ms35t8652.
