# Supplementary material for: LightBox: A multiwell plate illumination system for photoactive molecule characterization
Source: J Biophotonics. 2021 Feb 22;14(5):e202000481. doi: 10.1002/jbio.202000481 (PMC11475348; doi:10.1002/jbio.202000481)
Supplement: Supplementary file 1 — Appendix S1: Supporting Information [file JBIO-14-e202000481-s001.docx]

Supporting Information

LightBox: a multiwell plate illumination system for photoactive molecule characterization Supporting Information

A. D. Bounds^1^*, R. D. Bailey^1^, C. T. Adams^2^, D.C. Callaghan^2^, J. M. Girkin^1^

–––––––––

^1^ Centre for Advanced Instrumentation, Department of Physics, Durham University, South Road, Durham, DH1 3LE, United Kingdom

^2^ Department of Biosciences, Durham University, South Road, Durham, DH1 3LE, United Kingdom

*Correspondence: A.D. Bounds, Centre for Advanced Instrumentation, Department of Physics, Durham University, South Road, Durham, DH1 3LE, United Kingdom

Email: a.d.bounds@durham.ac.uk

**A. Pulse Width Modulation**

The PWM duty cycle i.e. the illumination intensity can be set using an internal resistor mounted on the electronics box front panel, which controls the output of a pulse generator chip (Texas Instruments, 555). Alternatively, the PWM can be obtained directly from an external TTL pulse generator provided via BNC by the control box. The LED current is modulated between on-off states at a frequency of 1 kHz, which is much slower than typical chemical processes but much faster than the response of the human eye and typical exposure times. A LED driver chip (ON Semiconductor, CAT4101) that is suitable for use with PWM is used to control the drive current. The LED drive current is set by a trim-pot, allowing tuning to different LED current limits should different LEDs be used.

**B. Control program sensor monitoring**

The RPi provides a +3 V supply to both the temperature sensor and a low-pass zero-bias PCB on which the photodiode is mounted. The photodiode low-pass zero-bias circuit has a time constant of approximately 1 second, much longer than the 1 ms PWM timescale, and thus averages over the PWM to obtain an average illumination intensity. Both sensors produce voltages that are converted to digital inputs on the RPi using an analog-to-digital converter (Analog Devices TMP37FT9Z). The voltages from the temperature sensor and photodiode are linearly proportional to the box temperature and illumination intensity and are recorded to a comma-separated variable (CVS) file on a USB stick inserted into RPi within the control box. Readings are taken every 5 seconds and saved with the sample name and the date and time that the sequence begun. The intensity measured by the monitor photodiode is lower by a factor measured as 1.4 compared to the intensity measured at the multiwell plate, a reduced intensity at the photodiode position is expected given that the photodiode is perpendicular to the illumination axis and is corrected for before being saved.

**C. Cell cultures and staining**

FDA/PI staining is a standard technique in biology with well-established cell culture protocols, so is only briefly described in this appendix. Four MWPs were used to demonstrate the LightBox and characterize the effectiveness of the photosensitizer. Two MWPs were used for FDA staining and two for PI staining, with one of each illuminated by the LightBox and one of each used as a control that is not illuminated. Black well, clear bottom 96 well plates were used. The experiment was conducted over three days.

On Day 1, 24 mL of HACAT cell culture was used to seed the MWPs, resulting in 100 µL of cell culture per well, approximately 20,000 cells per well. Cell culture comprised of DMEM GlutaMAX (Gibco product #31966021), 10% fetal bovine serum (Gibco product #10270106) and 1% Penicillin-Streptomycin (Gibco product #15070063).

On Day 2, 100 µL of the compound solution was administered to the MWPs, with different compound concentrations administered to different wells according to Figure 5(a).

The MWP was incubated for 1 hour at 37°C before exposure to the LightBox for 300 seconds at 100% intensity (0.23 mW/mm^2^), after which, the MWP was incubated for a further 24 hours at 37°C, 5% CO_2_.

On Day 3 the cell culture medium was then removed and rinsed with PBS before being stained with FDA or PI for 10 minutes at room temperature. The staining solution was then removed and the cell culture medium washed with PBS. Sample fluorescence was assessed with a fluorescent plate reader illuminating the sample at 535 nm and detecting fluorescence at 485 nm for PI stains, and illuminating and detecting at 485 nm and 520 nm for FDA stains. Standard deviations are calculated from the six repeats at each concentration.
